# Subjective cognitive decline—a neglected but preventable public health concern: development and validation of a risk prediction model for subjective cognitive decline in older adults: a cross-sectional survey study from Anhui Province in eastern China

**DOI:** 10.3389/fpubh.2025.1665324

**Published:** 2025-10-23

**Authors:** Huan Liu, Xinyu Hu, Qingwei Liu, Jiahui Min, Yang Luo, Jun-kai Dou, Qin Xu, Xiubin Tao, Ming Zhang

**Affiliations:** ^1^Department of Hemodialysis, The First Affiliated Yijishan Hospital of Wannan Medical College (Yijishan Hospital of Wannan Medical College), Wuhu, Anhui, China; ^2^Department of Cardiovascular, The Second Affiliated Hospital of Wangnan Medical College, Wuhu, Anhui, China; ^3^Department of Nursing, Shandong Provincial Hospital Affiliated to Shandong First Medical University (Shandong Provincial Hospital), Jinan, China; ^4^School of Public Health, Wannan Medical College, Wuhu, Anhui, China; ^5^School of Clinical Medical, Wannan Medical College, Wuhu, Anhui, China; ^6^Department of Nursing, Lu’an Hospital of Anhui Medical University, Luan, Anhui, China; ^7^Department of the Interventional, The First Affiliated Hospital of Wannan Medical College (Yijishan Hospital of Wannan Medical College), Wuhu, Anhui, China; ^8^Department of Nursing, The First Affiliated Yijishan Hospital of Wannan Medical College (Yijishan Hospital of Wannan Medical College), Wuhu, Anhui, China; ^9^School of Innovation and Entrepreneurship, Wannan Medical College, Wuhu, Anhui, China; ^10^Key Laboratory of Philosophy and Social Science of Anhui Province on Adolescent Mental Health and Crisis Intelligence Intervention, Hefei Normal University, Hefei, China

**Keywords:** older adults, subjective cognitive decline symptoms, cross-sectional study, prediction model, China

## Abstract

**Background:**

Subjective Cognitive Decline (SCD) is a significant risk factor for dementia and is prevalent among older adults in China. This study aimed to assess the prevalence and associated factors of SCD among older adults in Anhui Province, and to develop a validated risk prediction model.

**Methods:**

A cross-sectional study was conducted from July to August 2024 involving 3,124 older adults from Anhui Province. Data were collected using the Subjective Cognitive Decline Questionnaire (SCD-Q9), the FRAIL scale, the Geriatric depression scale-5(GDS-5), the Lubben Social Network Scale-6 (LSNS-6), and the Mini Nutritional Assessment Short Form (MNA-SF). Predictive factors were identified through univariate and multivariate analyses. A logistic regression model was used to identify SCD correlates, and a nomogram was developed. Model performance was evaluated using calibration curves, ROC-AUC, and decision curve analysis (DCA).

**Results:**

The prevalence of subjective cognitive decline among the older adults in Anhui Province was 69.1% (2,158/3124). Binary logistic regression analysis showed that, 70–79(OR = 1.306, 95% CI 1.081–1.576), and 80-89(OR = 1.434 95% CI 1.054–1.950), have been hospitalized in the past year (OR = 1.424, 95% CI = 1.202–1.686), frail (OR = 2.140, 95% CI = 1.689–2.712), malnutrition (OR = 2.157, 95% CI = 1.806–2.576), depression symptom(OR = 2.500, 95% CI = 2.031–3.077), social isolation (OR = 1.759, 95% CI = 1.420–2.180) were significantly associated with subjective cognitive decline.

**Conclusion:**

The developed nomogram provides a reliable tool for predicting SCD risk in older adults, supporting early screening and intervention in clinical practice.

## Introduction

According to the data from the seventh national population census conducted by the National Bureau of Statistics of China in 2020, the population aged 60 and above in mainland China has reached 264.02 million, accounting for 18.7% of the total population, among which the population aged 65 and above is 190.64 million, accounting for 13.5% of the total population. China has entered a stage of rapid aging development (1, 2). With the significant acceleration of China’s aging process, the concept of healthy aging was introduced to China in the 1990s, leading to a heightened focus on the health issues of the older adults. As a severe geriatric syndrome, subjective cognitive decline has gradually garnered widespread attention (3). In this context, subjective cognitive decline, an age-related geriatric syndrome, has become one of the prominent public health issues worldwide (4). Subjective cognitive decline (SCD) refers to the self-reported experience of a significant decline in an individual’s memory and/or other cognitive abilities relative to previous performance levels in the absence of objective neuropsychological deficits (5). Subjective cognitive decline not only seriously damages the cognitive function of the older adults, reduces the quality of life, increases hospitalization and mortality rates, but also brings a heavy economic burden to the older adults themselves, their families, society, and the health care system. A growing body of evidence suggests that individuals at risk of SCD have a significantly increased risk of developing cognitive dysfunction and dementia in the future (6, 7). In light of this, SCD has been regarded as the clinically asymptomatic preclinical stage of AD and is considered the first presenting symptom of AD (8).

Research has identified that individuals experiencing SCD may have subtle yet detectable cognitive impairments that traditional screening tests, such as the Mini-Mental State Examination, cannot capture effectively (9). Consequently, this can contribute to significant long-term consequences, as individuals may not receive the necessary diagnostic attention or interventions until more severe cognitive impairment has manifested. Furthermore, there’s growing evidence illustrating that SCD is linked to an increased risk of progressing to more severe cognitive disorders, such as Mild Cognitive Impairment (MCI) and dementia (10). For instance, a study highlighted that individuals with SCD exhibited an annual conversion rate to dementia of 1.12%, which is notably higher than the 0.45% observed in healthy controls (11). This escalated risk suggests that those reporting SCD may represent a vulnerable group that, despite appearing cognitively intact through objective measures, maybe on a trajectory toward cognitive decline. In addition to its predictive qualities, subjective cognitive decline is also associated with various psychosocial factors. Individuals reporting SCD often also exhibit symptoms of anxiety and depression, which may impair their quality of life and exacerbate their perception of cognitive dysfunction (12). For example, the presence of anxiety or depressive symptoms significantly correlates with SCD, indicating that mood disorders can influence self-reported cognitive difficulties (13).

Given the current absence of effective drugs and treatments worldwide that can significantly slow the progression of dementia in the older adults, primary prevention and early targeted interventions for subjective cognitive decline in older adults have become crucial measures in controlling its increasing prevalence. Currently, there are no reports on predictive models for subjective cognitive decline in the older adults. This study aimed to identify factors associated with subjective cognitive decline in older adults and to construct a nomogram based on a model predicting subjective cognitive decline in the older adults. On this basis, this study constructed a comprehensive risk prediction model for subjective cognitive decline for the first time for older adults in Anhui Province based on the database of the Anhui 2024 Cross-Sectional Study of Older Adults’ Mental Health and Behavior. In addition, this study innovatively applied the tool of a nomogram. Nomogram can effectively quantitatively integrate the predicted values of multiple variables, providing clinicians with a clear and intuitive risk assessment tool, enabling them to quickly and easily assess the risk of subjective cognitive decline, and then quickly and accurately identify high-risk populations, which can provide a relatively powerful aid in the identification and prevention of the risk of subjective cognitive decline in older adults.

A cross-sectional study conducted by Feng L et al., aimed at investigating the potential association between cognitive decline and nutrition in Chinese older adults, indicated that participants with a higher risk of malnutrition and poorer nutritional status were more likely to experience cognitive decline (14). A longitudinal study highlighted the importance of nutritional status in older adults experiencing SCD. The findings suggested that factors such as body mass index (BMI), dietary intake, and nutritional assessments (like the Mini-Nutrition Assessment) were significantly associated with clinical progression in patients, indicating that poorer nutritional status correlated with higher risks of cognitive decline and transitions from SCD to MCI or Alzheimer’s disease. The study observed that individuals with lower BMI and poorer nutritional assessments had elevated risks of clinical progression, suggesting that maintaining a healthy diet could potentially mitigate the risks of cognitive decline in the cohort (15). Additionally, nutritional inadequacies, such as micronutrient deficiencies, were identified as risk factors associated with SCD. Mainly, older adults exhibiting malnutrition were found to have a higher prevalence of cognitive decline. This malnutrition is often linked to insufficient intake of essential nutrients that are critical for maintaining cognitive health, such as vitamins and minerals. Infections, lack of appetite, and socio-economic factors often exacerbate malnutrition in older populations, further contributing to cognitive challenges (14, 16). The complexities surrounding the relationship between nutrition and cognitive health also suggest that inflammation could play a role in cognitive dysfunction and undernutrition. The aging process is often accompanied by increased oxidative stress and inflammation, hindering cognitive processes. Suitable dietary interventions, therefore, could address nutritional deficiencies and combat underlying inflammatory responses affecting cognitive health (17, 18).

Cognitive dysfunction and frailty are interrelated, with cognitive dysfunction being relatively prevalent among individuals experiencing frailty (19). The mechanisms linking SCD and frailty seem to be complex and multifactorial. Factors such as inflammation, nutritional status, and chronic illnesses may contribute to both SCD and frailty conditions by accentuating the decline in physiological reserves (20, 21). Additionally, studies show that the presence of frailty is predictive of future cognitive decline, reinforcing the idea of a bidirectional relationship where cognitive status can influence physical health and vice versa (22).

A survey of 1,163 older adults in the community revealed a close relationship between subjective cognitive decline and depression, as well as objective cognitive levels (23). Moreover, a population-based study on cognitively unimpaired adults around 70 demonstrated similar associations. In this context, higher levels of SCD were observed, particularly in individuals reporting symptoms of anxiety and depression (24). Lee et al. (25) found that the degree of SCD in older adults is significantly associated with their depressive symptoms and perceived health status. Additionally, when the older adults experience depression and anxiety, it increases the likelihood of cognitive decline (26). Lee et al. found that subjective cognitive decline can trigger negative emotions such as anxiety and depression, reduce the quality of life in the older adults, and may increase the risk of cognitive impairment (27).

Social isolation has been defined as the objective lack or paucity of social contacts and interactions with family members, friends, or the wider community (28). Research has found that older adults are susceptible to the effects of social isolation, which is often associated with adverse health outcomes such as cognitive (29) and functional decline (30), cardiovascular diseases (31), and increased risk of mortality (32). Studies have shown that low levels of social support are a risk factor for SCD in older adult community members (33). Older adults with low social support participate less in social activities, receive less brain stimulation, and lack emotional communication and spiritual sustenance, leading to depression. Livingston G, et al. reported in The Lancet highlights that social isolation in later life is one of the key modifiable risk factors for dementia in older adults (34). To sum up, we hypothesize that subjective cognitive decline is associated with frailty, malnutrition, depression, and social isolation.

There is currently no predictive model developed using large-sample, multicenter cross-sectional studies to identify older adults at high risk of subjective cognitive decline. Risk prediction models are crucial tools for assessing the risk of subjective cognitive decline in older adults, as they integrate the predictive values of multiple variables, enabling the early identification of individuals with subjective cognitive decline and the formulation of targeted early intervention strategies. Previous studies have primarily focused on the prevalence and influencing factors of subjective cognitive decline; however, effective tools for quantitatively assessing the risk of subjective cognitive decline in older adult patients remain relatively scarce. Therefore, this study aims to identify risk factors associated with subjective cognitive decline in the older adults and utilize these risk factors to develop a nomogram-based prediction model for assessing the risk of subjective cognitive decline in the older adults, thereby providing a reference for the formulation of early intervention strategies and the management of older adult health. Based on the above understanding, in this large-sample, multicenter, cross-sectional study, we examine the associations between social isolation, depressive symptoms, malnutrition, frailty, and subjective cognitive decline among the older adults in Anhui Province.

### Measures

Our research team used the convenience sampling method in the cross-sectional study to survey the older adults in Anhui Province, China, from July to August 2024. This provincial-level large-sample cross-sectional study called the “2024 Anhui Province older adults Psychological and Health Promotion Study” was conducted in 16 cities in Anhui Province, China, and was organized and implemented by the Yugong Nursing Research Design and Data Analysis Team of Yijishan Hospital of Wannan Medical College.

### Participants

The inclusion criteria for participants in this study are: (1) age ≥ 60 years old; (2) citizens of Anhui Province; (3) individuals who can complete the questionnaire themselves or with the assistance of the researcher. Exclusion criteria are (1) participants who have been diagnosed with severe mental disorders or neurological diseases by a second-level hospital or above; (2) those who have cognitive impairment and unable to cooperate in completing the questionnaire; (3) older adults who are in the terminal stage of the disease; (4) has severe visual or hearing impairment.

### Data collection methods

The investigation team consisted of 2 nursing graduate students and 100 undergraduate students from Wannan Medical College. Before the investigation, all investigators should receive standardized training and unified questionnaire survey methods and techniques. Investigators can conduct investigations only after they have mastered the skills and passed the assessment to ensure the accuracy of the investigation. Before the investigation, the purpose and significance of this study were explained to the older adults. After obtaining consent from the older adults and obtaining oral informed consent, the investigator dictated the contents of the questionnaire one by one and assisted the older adults to fill in the questionnaire based on the respondents’ own answers. Each questionnaire was completed on average. The time is 10 to 15 min. All questionnaires are distributed on site and automatically recycled after completion to ensure the completeness and accuracy of the data. A total of 3,188 questionnaires were distributed this time, and 3,124valid questionnaires were recovered, with an effective recovery rate of 98.0%.

## Measurements

### Demographic characteristics

The demographic questionnaire includes gender, age, education level, residential area, whether to drink alcohol (yes/no), whether to smoke (yes/no), whether to have a spouse (yes/no), whether to have chronic diseases (yes/no), and whether to undergo annual health check-ups (yes/no).

### The subjective cognitive decline

The subjective cognitive decline was measured using the Subjective Cognitive Decline Questionnaire (SCD-Q9). The SCD-Q9 was originally developed by Gifford et al. in the United States in 2015 (35). Hao et al. had demonstrated that the Chinese version of the SCD-Q9 has good internal reliability and validity, and has been widely utilized for the screening of SCD among the older adults in China (36). The total score of the SCD-Q9 scale is distributed from 0 to 9, with higher scores indicating more severe SCD symptoms. Therefore, participants were divided into the following two categories: non-subjective cognitive decline (SCD-Q9 score<3), and subjective cognitive decline (SCD-Q9 score≥4). In this study, the Cronbach’ s alpha of the 8-item subjective cognitive decline scale was 0.87.

### Frailty

Frailty was measured using the FRAIL scale (37). Compiled by the International Society of Nutrition and Aging, it includes 5 questions: fatigue, loss of endurance, limited free movement, existing diseases, and weight loss. Each item is scored 0 or 1; the maximum score is 5 points, and a test result of 3 or greater indicates a higher risk of frailty. The validity of this scale has been verified among Chinese community-dwelling older adults (38). In this study, the Cronbach’s coefficient of the scale in this study was 0.84.

### Depressive symptom

Depressive symptom was measured using the Geriatric depression scale-5(GDS-5). The scale is a depressive symptom assessment tool specifically for older adults. It was simplified by American scholar Hoy (39) and others in 1999 based on the long version of the Geriatric Depression Scale. The scale has 5 items in total, each item is scored as 0 = no, 1 = yes, with a total score of 0 to 5 points; the higher the score, the more severe the individual’s depressive symptoms are; the score≥2 points represent the presence of depressive symptoms (40), the Cronbach’s coefficient of the scale in this study was 0.76.

### Social isolation

Social isolation was measured using the Lubben Social Network Scale-6 (LSNS-6). The scale was compiled by Lubben et al. (41). It consists of two dimensions: family network and friend network. It has 6 items in total. The score of a single item is 0 to 5. It is based on the family members and friends that the older adults have contacted or come into contact with in the past month. Quantity is used as a basis to evaluate the social isolation status of the older adults. The numbers 0, 1, 2, 3 to 4, 5 to 8, and≥9 are assigned values 0 points, 1 point, 2 points, 3 points, 4 points, and 5 points, respectively. The LSNS-6 total score is 0–30 points; the higher the score, the more active the social network of the older adults, and a total score ≥12 points is considered social isolation (42). The Cronbach’s a coefficient of this scale in this study was 0.75.

### Nutritional status

The nutritional status of older adults in the study was assessed using the Mini Nutritional Assessment Short Form (MNA-SF) (43), which is a very short questionnaire used to quickly assess an individual’s nutritional status. It includes 6 questions, focusing on loss of appetite, weight loss, psychological impact from acute disease stress, mobility (bedridden or wheelchair-bound), mental and psychological problems, and BMI in the past 3 months. The total score of the MNA-SF ranges from 0 to 14, with 0–7 indicating malnutrition, 8–11 indicating risk of malnutrition, and 12–14 indicating normal nutritional status. In this study, the nutritional status of the older adults was divided into two categories—normal (MNA-SF total score≥12 points) and poor nutritional status (MNA-SF total score <11 points) for analysis (44). The Cronbach’s a coefficient of this scale in this study was 0.81.

### Statistical analysis

In this study, all continuous variables were expressed as mean and standard deviation, while categorical variables were presented as frequency (n) and percentage (%). Characteristics of older adults were compared by subjective cognitive decline using the χ^2^ test to obtain statistical significance. Pearson correlation analysis was used to explore the correlation between frailty, malnutrition, depressive symptoms, social isolation and subjective cognitive decline. Only parameters that showed a significant difference (*p* < 0.05) based on univariate analysis were selected for binary logistic regression analysis. After conducting binary logistic regression analysis, a nomogram with a concordance index (c-index) was constructed using R software. The model performance was evaluated using ROC curves, calibration curves, and AUC. The validity of the model fit was assessed by performing the Hosmer-Lemeshow goodness-of-fit test (Hosmer-Lemeshow statistic ≥ 0.05). Predictors’ clinical benefits were compared using decision curve analysis (DCA) curves. All analyses in this study were conducted using R 4.4.1 software.^1^ All tests were two-tailed, and a *p*-value of less than 0.05 was considered statistically significant.

## Results

### Description of the sociodemographic characteristics of the older adults

In this cross-sectional study, a total of 3,124 older adults were ultimately included. Table 1 presents the demographic characteristics of the older adults. Among the 3,124 older adults, 52.3% (n = 1,634) were male and the rest were female, 47.7% (*n* = 1,490). The age range of the older adults was from 60 to 99 years old， with a mean of 69.86 ± 7.89. The places of residence were 977(31.9%), 761(24.9%), and 1,323 (43.2%) for county, town, and city, respectively. Detailed sociodemographic characteristics of participants are presented in Table 1.

**Table 1:** Univariate analysis of the participants’ demographic.

Factors		No-subjective cognitive decline (*n* = 966, 30.9%)	Subjective cognitive decline (n = 2,158, 69.1%)	χ^2^	*p* value
Gender	Male	496 (30.4%)	1,138 (69.6%)	0.52	0.47
	Female	470 (31.5%)	1,020 (68.5%)		
Social isolation	No	823 (35.9%)	1,471 (64.1%)	99.22	< 0.001
	Yes	143 (17.2%)	687 (82.8%)		
Depressive symptoms	No	807 (40.8%)	1,169 (59.2%)	247.64	< 0.001
	Yes	159 (13.9%)	989 (86.1%)		
Malnutrition	No	482 (50.6%)	470 (49.4%)	248.99	< 0.001
	Yes	484 (22.3%)	1,688 (77.7%)		
Frailty	No	856 (37.7%)	1,412 (62.3%)	180.27	< 0.001
	Yes	110 (12.9%)	746 (87.1%)		
Hospitalization in the past year	No	611 (34.6%)	1,153 (65.4%)	26.12	< 0.001
	Yes	355 (26.1%)	1,005 (73.9%)		
Age	60–69	634 (34.8%)	1,186 (65.2%)	34.76	< 0.001
	70–79	247 (27.0%)	668 (73.0%)		
	80–89	69 (22.3%)	241 (77.7%)		
	≥90	16 (20.3%)	63 (79.7%)		
Place of residence	City	315 (31.4%)	688 (68.6%)	0.24	0.89
	Town	235 (30.3%)	540 (69.7%)		
	Countryside	416 (30.9%)	930 (69.1%)		
The location in Anhui	North Anhui	427 (30.4%)	979 (69.6%)	0.38	0.83
	Central Anhui	279 (31.2%)	614 (68.8%)		
	Southern Anhui	260 (31.5%)	565 (68.5%)		
Education	Illiterate	338 (29.3%)	816 (70.7%)	6.36	0.50
	Elementary school	226 (29.6%)	537 (70.4%)		
	Junior school	146 (32.2%)	307 (67.8%)		
	High school	92 (33.8%)	180 (66.2%)		
	vocational school	52 (34.0%)	101 (66.0%)		
	Associate degree	30 (31.6%)	65 (68.4%)		
	undergraduate	50 (36.5%)	87 (63.5%)		
	master’s degree or above	32 (33.0%)	65 (67.0%)		
Marital status	Married	711 (32.1%)	1,505 (67.9%)	6.14	0.29
	No Longer Living Together	39 (28.5%)	98 (71.5%)		
	Divorced	36 (30.0%)	84 (70.0%)		
	Widowed	126 (26.5%)	349 (73.5%)		
	Unmarried	27 (30.7%)	61 (69.3%)		
	Others	27 (30.7%)	61 (69.3%)		
Smoking	No	539 (32.1%)	1,141 (67.9%)	2.40	0.30
	quit smoking	271 (29.9%)	636 (70.1%)		
	Yes	156 (29.1%)	381 (70.9%)		
Drinking	No	457 (32.3%)	960 (67.7%)	2.19	0.33
	abstain from alcohol	269 (29.6%)	640 (70.4%)		
	Yes	240 (30.1%)	558 (69.9%)		

### Factors associated with subjective cognitive decline in the chi-square test

In this study, the prevalence of subjective cognitive decline among the older adults was 69.1% (2158/3124). Significant differences were observed in social isolation, depressive symptoms, risk of malnutrition, frailty, hospitalization in the past year, and age groups among older adults with subjective cognitive decline (*p* < 0.05, Table 1).

**Figure 1:**
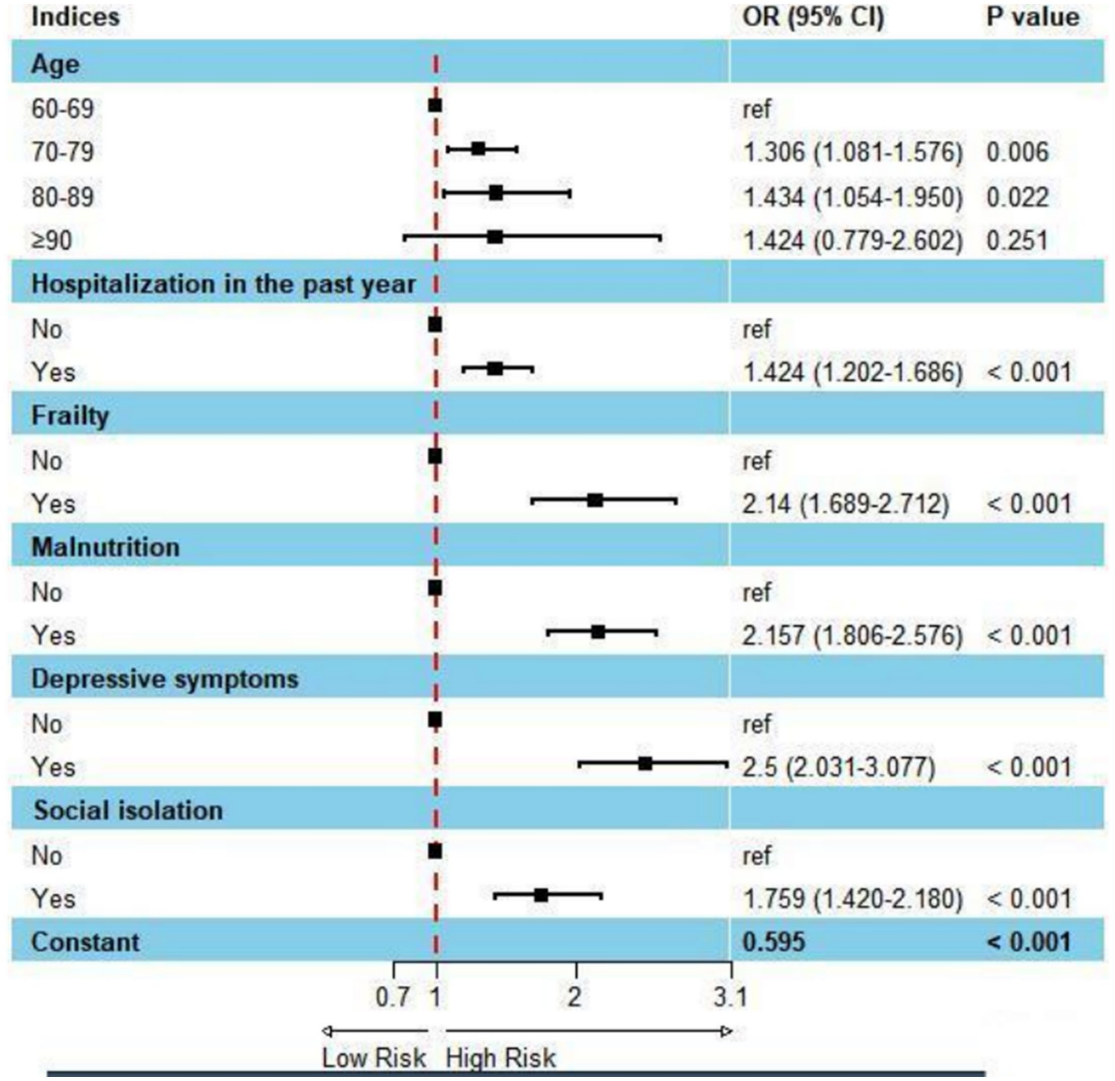
Forest plot: factors affecting subjective cognitive decline using binary logistic regression analysis.

The risk factors influencing subjective cognitive decline in Chinese older adults are shown in Table 2. In binary logistic regression analysis, 70–79(OR = 1.306, 95% CI 1.081–1.576), and 80-89(OR = 1.434 95% CI 1.054–1.950) were significantly associated with subjective cognitive decline. When Chinese older adults with have been hospitalized in the past year (OR = 1.424, 95% CI = 1.202–1.686), frail (OR = 2.140, 95% CI = 1.689–2.712), malnutrition (OR = 2.157, 95% CI = 1.806–2.576), depression symptom(OR = 2.500, 95% CI = 2.031–3.077), social isolation (OR = 1.759, 95% CI = 1.420–2.180), they were at a higher risk of subjective cognitive decline (Figure 1).

**Table 2:** Influencing factor assignments.

Factors	Assignment
Age	60–69 = 1，70–79 = 2，80–89 = 3， ≥ 90 = 4
Hospitalization in the past year	No = 0，yes = 1
Frailty	No = 0，yes = 1
Malnutrition	No = 0，yes = 1
Depressive symptoms	No = 0，yes = 1
Social isolation	No = 0， yes = 1

### Predictive model development

**Figure 2:**
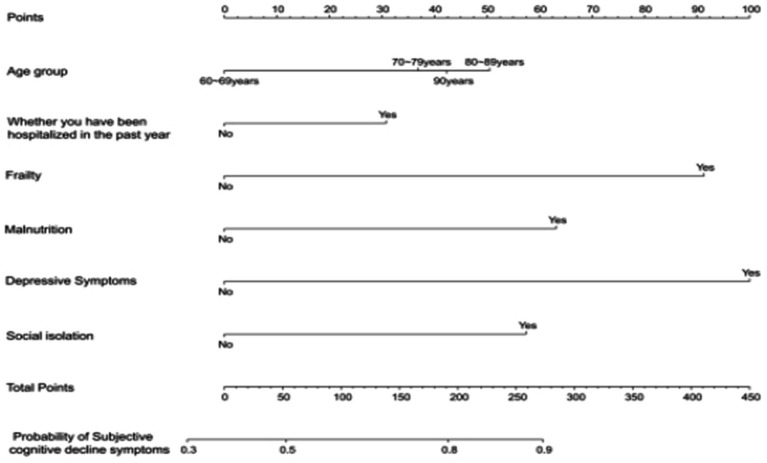
Nomogram used to quantitatively predict the risk of subjective cognitive decline in the older individuals.

By employing a stepwise selection method to screen predictive variables, variables with *p* < 0.05 were included in a multivariate binary logistic regression analysis, which identified the key variables for constructing the nomogram. After screening the predictive variables for the model of subjective cognitive decline in the older adults, we utilized the lrm function from the Design package in RStudio to build the model and visualized it using the “nomogram” function (Figure 2). According to the nomogram, depression was identified as the strongest predictor of subjective cognitive decline in the older adults, followed by malnutrition, frailty, age, social isolation, and hospitalization within the past year (Table 3).

**Table 3:** Binary logistic regression analysis of subjective cognitive decline.

Indices	β	Wald	*p* value	OR	95% CI
Age		11.543	0.009		
70–79	0.267	7.685	0.006	1.306	1.081–1.576
80–89	0.360	5.282	0.022	1.434	1.054–1.950
≥90	0.353	1.320	0.251	1.424	0.779–2.602
Hospitalization in the past year	0.353	16.703	< 0.001	1.424	1.202–1.686
Frailty	0.761	39.611	< 0.001	2.140	1.689–2.712
Malnutrition	0.769	71.844	< 0.001	2.157	1.806–2.576
Depressive symptoms	0.916	74.657	< 0.001	2.500	2.031–3.077
Social isolation	0.565	26.625	< 0.001	1.759	1.420–2.180
Constant	−0.519	38.965	< 0.001	0.595	

### Validation of the prediction model

**Figure 3:**
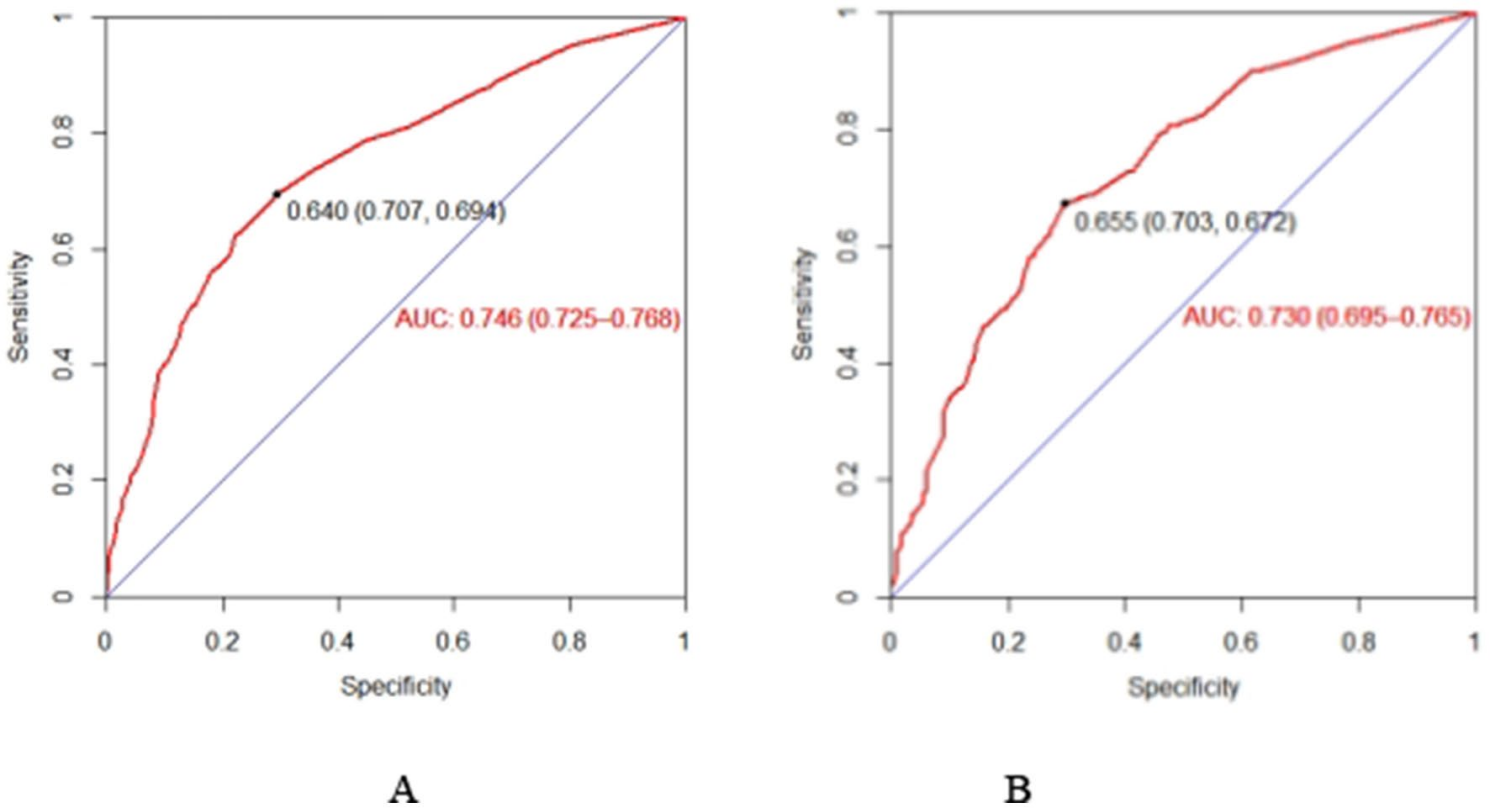
The AUC of the nomogram in the training set **(A)** and set **(B)**. ROC curve of the nomogram for quantitatively predicting the risk of subjective cognitive decline in older adults. **(A)** The ROC curve was generated using the training dataset. **(B)** The ROC curve was generated using the validation dataset.

The Bootstrap resampling method was employed to validate the constructed model, with 1,000 repeated samplings conducted for internal validation of the nomogram model. The C-index in the training set was 0.746 (95% CI: 0.728–0.770), and in the validation set, it was 0.739 (95% CI: 0.709–0.778), indicating that the model possesses good discriminative ability. Additionally, the AUC value was determined to assess the discriminatory capability of the nomogram. As illustrated in Figures 3A,B, the AUC for predicting subjective cognitive decline using the nomogram in the training set was 0.746 (95% CI: 0.725–0.768). In the validation set, the AUC was 0.730 (95% CI: 0.695–0.765). The aforementioned research data clearly demonstrate that the nomogram possesses a certain level of discriminative ability and predictive value, and that they can somewhat correctly identify the risk of subjective cognitive decline or non-subjective cognitive decline in older adults.

### Calibration of the prediction model

**Figure 4:**
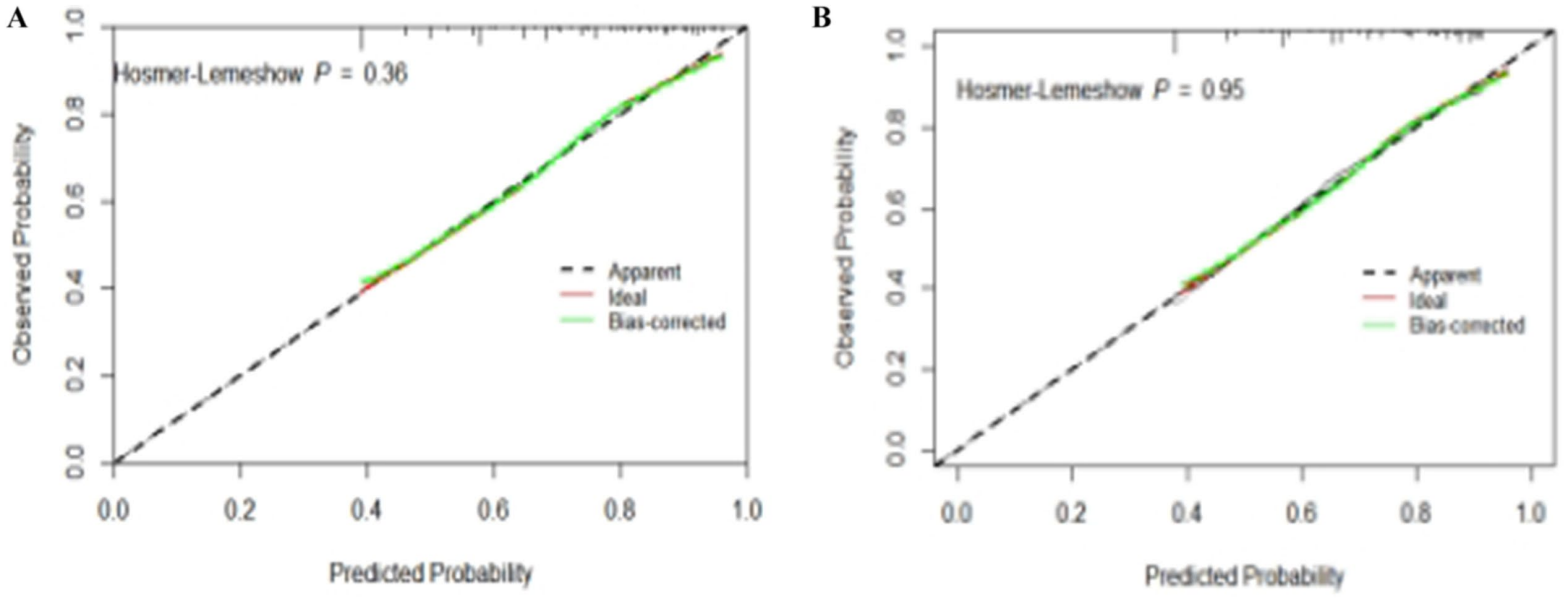
Calibration plots for quantitative prediction of subjective cognitive decline risk in older adults. **(A)** Calibration plot generated using training set data. **(B)** Calibration plot generated using validation set data.

The test results showed that the model fit well to both the training set (*χ*^2^ = 8.792, df = 8, *p* = 0.360) and the validation set (*χ*^2^ = 2.748, df = 8, *p* = 0.949). The calibration curves based on the multifactorial logistic regression model in Figures 4A,B demonstrate a high degree of consistency between the predicted and actual probabilities of subjective cognitive decline in the older adults, both in the training and validation sets.

### Assessment of clinical effectiveness

**Figure 5:**
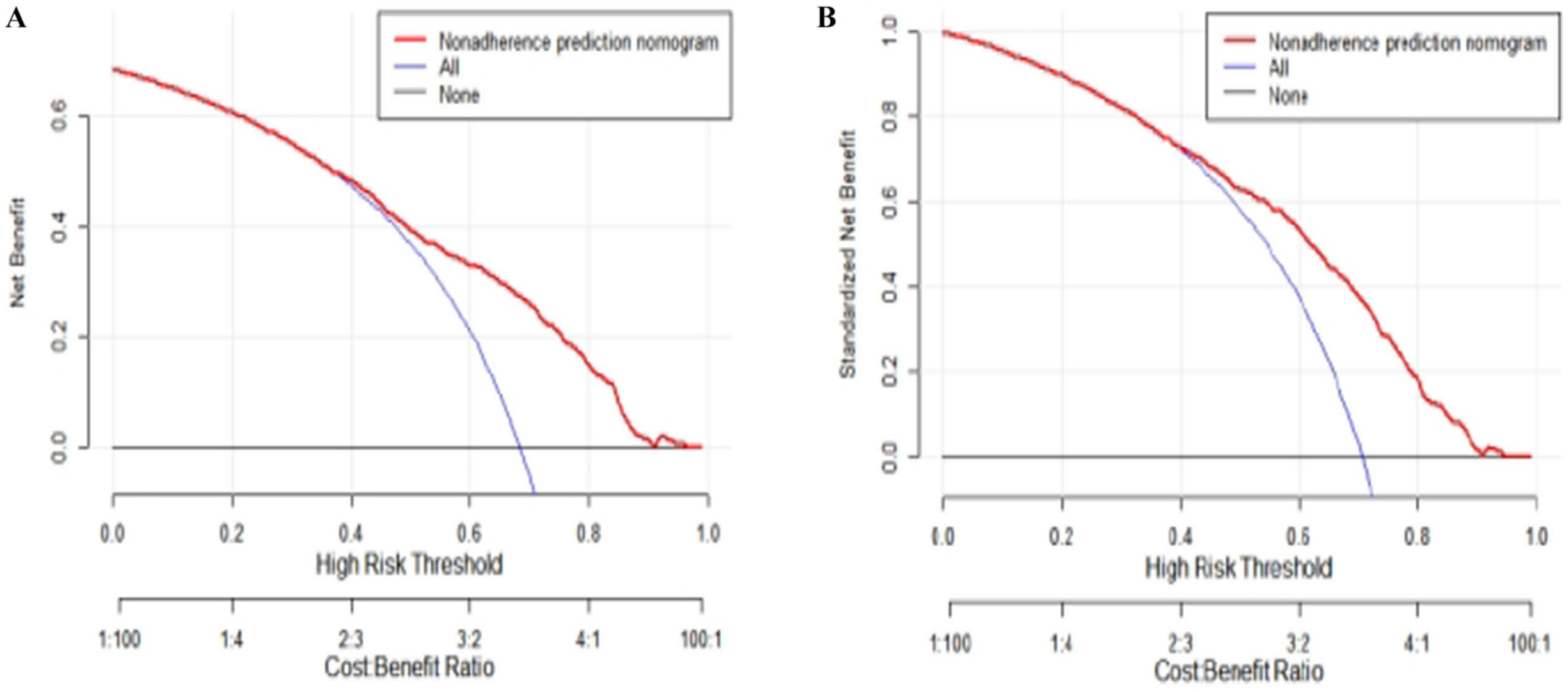
Decision curve analysis (DCA) plots for quantitatively predicting the risk of subjective cognitive decline in the older adults. **(A)** DCA plot generated using the training set data. **(B)** DCA plot generated using the validation set data.

The results of DCA indicate that the nomogram can yield significant net benefits for older adults experiencing subjective cognitive decline. In the training set, these benefits were observed within a risk threshold probability range of 38 to 90% (Figure 5A), and in the validation set, they were observed within a risk threshold probability range of 40 to 88% (Figure 5B).

## Discussion

The findings of this study indicated that the prevalence of subjective cognitive decline (SCD) among older adults is 69.1%, higher than in previous studies (45, 46). There may be several reasons for this outcome: (1) This discrepancy may be closely related to various factors such as region, economic level, education level, and lifestyle. As a region with relatively lagging economic development in China, the health status of the older adults in Anhui Province may be jointly influenced by a variety of complex factors, including socio-economic transformation, uneven distribution of medical resources, and cultural background. These factors may collectively contribute to the higher prevalence of SCD in this region. (2) The difference in cultural background and health beliefs may be a key factor. In traditional Chinese culture, the stigmatization of cognitive decline may be relatively weaker, or the perception of “forgetfulness” may be more common and accepted, which could encourage the older adults to more willingly report subjective cognitive changes. In contrast, within the context of Western culture, a strong fear of dementia may lead individuals to deny or downplay early symptoms. (3) The differences in social support structures and exposure to risk factors cannot be overlooked. Older adults in rural areas of China may face a higher risk of social isolation (e.g., due to labor migration leading to the “empty nest” phenomenon) and specific patterns of malnutrition (such as insufficient intake of micronutrients), which are known risk factors for SCD and frailty. (4) The higher prevalence of comorbidities (such as hypertension and diabetes) in this study cohort may also contribute to the higher prevalence of SCD. (5) Lastly, methodological considerations, such as sampling strategies, data collection methods, and the specific items and cutoff values of the SCD assessment tools used, may also be technical reasons contributing to the rate discrepancies.

Research indicates that individuals with subjective cognitive decline (SCD) have a significantly higher risk of progressing to mild cognitive impairment (MCI) and Alzheimer’s disease (AD) compared to those without SCD (47, 48). Therefore, the high prevalence of SCD among the older adults in Anhui Province may indicate a further increase in the burden of dementia in the region in the future. The findings of this study hold significant public health implications and underscore the urgency and importance of enhancing cognitive health interventions and early screening for the older adults in Anhui Province and similar regions.

This current study revealed that old adults who advanced age had a higher risk of developing subjective cognitive decline, which is consistent with that of Wang Nianfen (49) and Xue Chao (50). Wang et al. (49) suggested that the risk of subjective cognitive decline increases with age in the older adults, with being over 65 years old identified as an independent risk factor for both subjective cognitive decline and mild cognitive impairment. Moreover, the majority of subjective cognitive decline cases are not caused by diseases. Specifically, as age advances, the physiological functions of the older adults deteriorate, primarily including telomere dysfunction, aging of the adaptive immune system, decline in skeletal muscle mass and function, and cognitive decline. Therefore, subjective cognitive decline is a result of normal aging in the older adults (51).

Advanced age has been identified as a catalyst for chronic inflammation, and older adults often experience impaired physical function and reduced muscle mass (52). This heightened inflammatory state may have serious adverse effects on cognitive functions, particularly memory and executive functions, thereby increasing the risk of subjective cognitive decline in the old adults (53). Therefore, subjective cognitive decline is prevalent among the older adults, and healthcare professionals should pay more attention to the cognitive function status of individuals aged 65 and above. Additionally, this study found that the difference in subjective cognitive decline between individuals aged 90 and above and those aged 60–70 was not significant in the regression analysis. This may be attributed to the relatively small number of participants aged 90 and above in this study. In subsequent research, we will further enhance the age stratification of participants to ensure the representativeness of the study population.

This study indicated that older adults hospitalized within the past year had a higher risk of developing subjective cognitive decline, which is consistent with the studies (54). The intersection of hospitalization and subjective cognitive decline (SCD) in older adults represents a critical yet underexplored area in neurocognitive research, with implications for both clinical management and long-term prognostic assessment. The relationship between SCD and hospitalization is further complicated by the interplay of psychological and physiological factors. Old adult inpatients, due to their advanced age, experience gradual degenerative changes in various organs, a decline in immune function, and a reduction in individual reserve capacity, which increases the risk of cognitive frailty (55). Being removed from familiar environments and social circles, older adult inpatients suddenly have significantly less interaction with the outside world, making them highly susceptible to decreased appetite, loneliness, and depression. Depression can lead to systemic dysfunction, a decline in immunity, and impairments in multidimensional cognitive functions such as attention and processing speed, thereby significantly increasing the risk of cognitive frailty (56). Additionally, older adult inpatients are prone to decreased appetite, which can result in malnutrition, a decline in immune function, and possible inflammatory responses in the body, ultimately further elevating the risk of cognitive frailty (57).

This study indicated that old adults with depressive symptoms had a higher risk of developing subjective cognitive decline, which is consistent with the studies. With the acceleration of China’s population aging process, the prevalence of depressive symptoms among the older adults has significantly increased, and has become a serious public health issue that has seriously affected their health. A longitudinal study conducted by Zhang Z et al. revealed that older adult patients with depression may face a higher risk of cognitive decline and brain atrophy, and alleviating depressive symptoms in the older adults was highly beneficial for delaying cognitive decline (58). Using data from the China Health and Retirement Longitudinal Study (CHARLS), Zhou L et al. found that older adults with depressive symptoms performed worse cognitively and were at greater risk of developing mild cognitive impairment compared to those without depressive symptoms (59). The more severe the depressive symptoms in the older adults are in their daily lives, the fewer opportunities they have to interact with the outside world, and the less external information stimulation and social activity engagement they can receive, which may exacerbate subjective cognitive decline in the older adults, thereby forming a vicious cycle of depressive symptoms-subjective cognitive decline-depressive symptoms. Another possible reason is that depressive symptoms can lead to negative emotions in the older adults, thereby reducing sleep quality. Prolonged poor sleep quality can induce cardiovascular and cerebrovascular diseases in the older adults, which in turn can further lead to varying degrees of cognitive impairment (60). The exact role of depressive symptoms in individuals with SCD remains unclear. Research has found that depression may induce cognitive decline by increasing inflammation, amyloid deposition, and the formation of neurofibrillary tangles, all of which may lead to impaired neuroplasticity and hippocampal damage (61, 62). Furthermore, depression has a significant impact on reducing the ability to live independently and is a leading cause of disability worldwide (63).

Our study found that the nutritional status of the older adults was significantly associated with SCD. Existing studies have found that a limited variety of food and reduced intake of some micronutrients are associated with an increased risk of disability (64). Hsu et al. conducted a long-term cohort study of older men and found that the risk of malnutrition was significantly associated with 3-year cognitive decline (OR: 2.07, 95% CI: 1.05–4.08, *p* = 0.036) (65). Previous studies have indicated that nutritional deficiencies in the older adults may impair or affect their cognitive functions. There are several plausible explanations for our findings. Research studies have reported that micronutrient deficiencies are associated with adverse outcomes in SCD (66, 67). It has also been found that malnutrition caused by micronutrient deficiencies and its accompanying adverse effects can impair human metabolism, induce immune activation and inflammation, and lead to neurodegenerative changes in AD and the formation of *β*-amyloid plaques through multiple pathways, resulting in impaired cognitive function of the brain (68, 69). During the life cycle, the deficiency of certain B vitamins, minerals, lipids, and antioxidants in the older adults may further exacerbate the decline in brain physiological functions, thereby increasing the vulnerability of brain cell DNA damage, which can lead to cognitive decline in the older adults (70). Innovatively, recent studies highlight the role of metacognitive deficits in SCD as a mediator of nutritional outcomes. Impaired awareness of cognitive decline may lead to neglect of dietary needs, while malnutrition-induced neurobiological changes (e.g., altered neuropeptide Y levels) may exacerbate SCD symptoms (71, 72). Based on the information provided by the findings of this study, we recommend increasing the intake of protein, fat, and vitamins in the diet to enhance the nutritional status of the older adults, which will help mitigate the risk of their subjective cognitive decline.

Our study also found that the frailty status of older adults is significantly associated with SCD-positive symptoms, which is consistent with previous research findings (73). Reports have indicated that frail patients exhibit significantly reduced cognitive function compared to non-frail patients. Frailty is associated with an increased risk of moderate cognitive impairment, and the more pronounced the frailty, the faster the decline in cognitive function (74). Even though the underlying mechanisms of frailty and cognitive impairment remain unclear, several studies have been conducted globally to show a strong positive correlation between frailty and cognitive impairment (75, 76). Frailty and subjective cognitive decline is closely related symptoms in the aging population. The relationship between subjective cognitive decline (SCD) and frailty in older adults represents a critical intersection of cognitive and physical health, with significant implications for early intervention and preventive strategies. Frail older people tend to be less likely to participate in social activities and have relatively little contact with others because of their health, both of which are important for slowing the decline of brain function and preventing dementia in older people (77). Clinically, the identification of SCD in frail older adults offers a window for early risk stratification and personalized interventions. For instance, individuals with SCD and pre-frailty exhibit poorer outcomes, including lower gait speed, reduced grip strength, and higher mortality rates, compared to those with either condition alone (78, 79). By implementing a dynamic monitoring mechanism specifically designed for frailty in the older adults, along with targeted frailty intervention measures, it is possible for older adults to achieve a notable improvement in cognitive function while delaying the progression of frailty. This approach not only helps maintain or restore their subjective cognitive function but also enhances their overall quality of life in later years.

The findings of this study indicate that low levels of social support are a risk factor for the occurrence of SCD in community-dwelling older adults, which is consistent with previous research (80, 81). Globally, social isolation among the older adults has become a prominent mental health issue and has rapidly spread in recent decades (82). Research has found that high social isolation (SI) among the older adults, due to retirement and widowhood, reduces emotional communication, leading to a decline in their cognitive abilities (83). Additionally, studies have revealed that social isolation in the older adults leads to loneliness and depression, which in turn exacerbate cognitive decline. Furthermore, previous research by Kim indicates that social isolation among the older adults not only limits their access to necessary information but also hinders their ability to obtain help when needed, resulting in negative perceptions of interpersonal relationships, which naturally lowers the level of cognitive function (84). The results of a study conducted by Jang Y, et al., involving 2061 older adults Korean Americans, indicated that social isolation is a significant risk factor in both the logistic regression models for objective cognitive impairment and subjective cognitive impairment (85). The various risk factors of SCD are not isolated but interconnected and mutually influential. Study (86) suggests that cognitive decline is preventable, and effective control of these risk factors can reduce the risk of disease by 40%, further demonstrating the necessity of actively managing the risk factors of SCD in the older adults. Participating in activities is the most direct way to connect with the outside world, which can promote brain function, effectively improve the orientation, attention, and language communication abilities of the older adults, and prevent and delay the occurrence and development of cognitive impairment (87, 88). Increased frequency of contact with children can reduce the incidence of cognitive impairment in the older adults. The parent–child relationship is an important social relationship for the older adults, which can meet their spiritual needs, alleviate negative emotions, and stimulate positive and healthy behaviors (89).

At the clinical practice level, the application of predictive models has significantly enhanced healthcare workers’ ability to identify subjective cognitive decline in the older adults early and improve the efficiency of targeted interventions. Early intervention in cognitive function can effectively delay the progression of cognitive impairment.

From a policy perspective, developing a predictive model for subjective cognitive decline in the older adults is of profound significance for the rational allocation and precise intervention of public health resources. This research highlights that modifiable factors such as frailty, malnutrition, depressive symptoms, and social isolation are prevalent among the older adults and closely associated with subjective cognitive decline. By integrating these critical factors, the predictive model can accurately identify high-risk populations, providing policymakers with a scientific basis for decision-making and ensuring that limited medical resources are prioritized for early screening and intervention in these groups.

## Strengths and limitations

To the best of our knowledge, this study is the first cross-sectional study to investigate the prevalence of subjective cognitive decline among older adults in Anhui Province with a large sample and to examine its association with frailty, malnutrition, depressive symptoms, and social isolation. In addition, all survey instruments used to measure study results (such as SCD-Q9, GDS-5 scores, etc.) were validated for the Chinese older adults. In this study, we successfully established and validated a nomogram model capable of predicting subjective cognitive decline in the older adults. Although the model demonstrated relatively good predictive ability, it inevitably has certain limitations. First, because the study was cross-sectional, it was not possible to establish a causal relationship between frailty, malnutrition, depressive symptoms, social isolation, and SCD. Second, subjective cognitive decline was assessed using subjective measures of self-report scales and not a clinical diagnostic instrument. In addition, the participants were from a specific region, Anhui Province, China, which limits the generalizability of the findings, and therefore the diversity of sample sources needs to be emphasized to ensure universal representation among older adults living in China.

## Conclusion

This cross-sectional study developed and validated a nomogram for predicting the risk of subjective cognitive decline in the older adults. The model facilitates the quantitative assessment of risk factors for subjective cognitive decline in the older adults and demonstrates excellent predictive accuracy. By evaluating the risk of subjective cognitive decline in each participant, the model provides clinicians with a reliable tool for formulating intervention strategies for subjective cognitive decline in the older adults, optimizing the allocation of healthcare resources, and improving the prognosis and quality of life of the older adults. Moreover, it can provide geriatric medical professionals with more in-depth insights into the progression of subjective cognitive decline in the older adults, thereby identifying potential risk factors for subjective cognitive decline, to formulate targeted preventive intervention strategies.

## Data Availability

The raw data supporting the conclusions of this article will be made available by the authors, without undue reservation.

## Footnotes

^1^ http://www.R-project.org

## References

[1] Liu Z Bu T Akpinar S Jabucanin B. The association between China’s economic development and the passing rate of national physical fitness standards for elderly people aged 60-69 from 2000 to 2020. Front Public Health. (2022) 10:857691. doi: 10.3389/fpubh.2022.857691 35359759 PMC8961805

[2] The Lancet. Population ageing in China: crisis or opportunity? Lancet. (2022) 400:1821. doi: 10.1016/S0140-6736(22)02410-2, PMID: 36436518

[3] Cheng CH Hsiao FJ Hsieh YW. Dysfunction of inferior parietal lobule during sensory gating in patients with amnestic mild cognitive impairment. Front Aging Neurosci. (2020) 12:39. doi: 10.3389/fnagi.2020.00039, PMID: 32158387 PMC7052059

[4] Liu H Zou L Zhou R Zhang M Gu S Zheng J. Long-term increase in cholesterol is associated with better cognitive function: evidence from a longitudinal study. Front Aging Neurosci. (2021) 13:691423. doi: 10.3389/fnagi.2021.691423, PMID: 34220488 PMC8248815

[5] Song Y Wu H Chen S Ge H Yan Z Xue C. Differential abnormality in functional connectivity density in preclinical and early-stage Alzheimer’s disease. Front Aging Neurosci. (2022) 14:879836. doi: 10.3389/fnagi.2022.879836, PMID: 35693335 PMC9177137

[6] Rabin LA Smart CM Amariglio RE. Subjective cognitive decline in preclinical Alzheimer’s disease. Annu Rev Clin Psychol. (2017) 13:369–96. doi: 10.1146/annurev-clinpsy-032816-045136, PMID: 28482688

[7] Wang X Wang Z Hu H Wang X-T Wang Z-T Hu H-Y. Association of subjective cognitive decline with risk of cognitive impairment and dementia: a systematic review and meta-analysis of prospective longitudinal studies. J Prev Alzheimers Dis. (2021) 8:277–85. doi: 10.14283/jpad.2021.27, PMID: 34101784 PMC12280825

[8] Lin Y Shan PY Jiang WJ Sheng C Ma L. Subjective cognitive decline: preclinical manifestation of Alzheimer’s disease. Neurol Sci. (2019) 40:41–9. doi: 10.1007/s10072-018-3620-y, PMID: 30397816

[9] Pavel A Matei V Paun R Tudose C. How “subjective” is subjective cognitive decline? Psychiatry Clin Psychopharmacol. (2022) 32:299–305. doi: 10.5152/pcp.2022.22506, PMID: 38764884 PMC11082589

[10] Ball HA Coulthard E Fish M Bayer A Gallacher J Ben-Shlomo Y. Predictors and prognosis of population-based subjective cognitive decline: longitudinal evidence from the Caerphilly prospective study (CaPS). BMJ Open. (2023) 13:e073205. doi: 10.1136/bmjopen-2023-073205, PMID: 37844990 PMC10582873

[11] Purri R Brennan L Rick J Xie SX Deck BL Chahine LM. Subjective cognitive complaint in Parkinson’s disease patients with normal cognition: canary in the coal mine? Mov Disord. (2020) 35:1618–25. doi: 10.1002/mds.28115, PMID: 32520435 PMC7722141

[12] Xu Y Warwick J Eramudugolla R. No clear associations between subjective memory concerns and subsequent change in cognitive function: the PATH through life study. Eur J Ageing. (2022) 19:1181–8. doi: 10.1007/s10433-022-00694-2, PMID: 36506667 PMC9729657

[13] Kasai M Meguro K. Patients with very mild dementia may confuse objective cognitive impairments with subjective physical health or quality of life: the Tome City project in Japan. Front Psychol. (2018) 9:533. doi: 10.3389/fpsyg.2018.00533, PMID: 29706921 PMC5906737

[14] Feng L Chu Z Quan X Zhang Y Yuan W Yao Y. Malnutrition is positively associated with cognitive decline in centenarians and oldest-old adults: a cross-sectional study. EClinicalMedicine. (2022) 47:101336. doi: 10.1016/j.eclinm.2022.101336, PMID: 35497066 PMC9046105

[15] Doorduijn AS de van der Schueren MAE van de Rest O de Leeuw FA Hendriksen HMA Teunissen CE. Nutritional status is associated with clinical progression in Alzheimer’s disease: the NUDAD project. J Am Med Dir Assoc, (2023), 24:638–644.e1. 33239240 10.1016/j.jamda.2020.10.020

[16] Wu SY Lee SC Yeh NH Wang CF Hung SY Wu SJ. Dietary characteristics of elders with frailty and with mild cognitive impairment: cross-sectional findings and implications from the nutrition and health survey in Taiwan 2014-2017. Nutrients. (2022) 14:5216. doi: 10.3390/nu14245216, PMID: 36558375 PMC9782975

[17] Jamka M Chrobot M Jaworska N Brylak J Makarewicz-Bukowska A Popek J. Comparison of eating habits, body composition, and densitometric parameters between subjects with normal cognitive function and mild cognitive impairment: an observational study. Nutrients. (2024) 16:644. doi: 10.3390/nu16050644, PMID: 38474772 PMC10934958

[18] Goshen A Goldbourt U Shohat T Shimony T Keinan-Boker L Gerber Y. Diet quality in relation to healthy ageing: The Israeli longitudinal study on aging (ILSA) - a study protocol. BMJ Open. (2019) 9:e024673. doi: 10.1136/bmjopen-2018-024673, PMID: 31005912 PMC6500277

[19] McAdams-DeMarco MA Tan J Salter ML Gross A Meoni LA Jaar BG. Frailty and cognitive function in incident hemodialysis patients. Clin J Am Soc Nephrol. (2015) 10:2181–9. doi: 10.2215/CJN.01960215, PMID: 26573615 PMC4670760

[20] Hsieh TJ Chang HY Wu IC Chen CC Tsai HJ Chiu YF. Independent association between subjective cognitive decline and frailty in the elderly. PLoS One. (2018) 13:e0201351. doi: 10.1371/journal.pone.0201351, PMID: 30071051 PMC6072005

[21] Andrade AQ Widagdo I Lim R Kelly TL Parfitt G Pratt N. Correlation of frailty assessment metrics in one-year follow-up of aged care residents: a sub-study of a randomised controlled trial. Aging Clin Exp Res. (2023) 35:2081–7. doi: 10.1007/s40520-023-02491-y, PMID: 37452224 PMC10520153

[22] Tang H Zhu H Sun Q Qin H Wang S. Transitions in the cognitive frailty states in community-living older adults: a 6-year prospective cohort study. Front Aging Neurosci. (2021) 13:774268. doi: 10.3389/fnagi.2021.774268, PMID: 34924997 PMC8672135

[23] Wu H Luo Y Yao G Zhang G Sun J Feng X. Latent categories of subjective cognitive decline and related factors in community elderly. Chin J Soc Med. (2025) 42:202–6.

[24] Pavisic IM Lu K Keuss SE James SN Lane CA Parker TD. Subjective cognitive complaints at age 70: associations with amyloid and mental health. J Neurol Neurosurg Psychiatry. (2021) 92:1215–21. doi: 10.1136/jnnp-2020-325620, PMID: 34035132 PMC8522456

[25] Lee J Sung J Choi M. The factors associated with subjective cognitive decline and cognitive function among older adults. J Adv Nurs. (2020) 76:555–65. doi: 10.1111/jan.14261, PMID: 31713894

[26] Vlachos GS Cosentino S Kosmidis MH Anastasiou CA Yannakoulia M Dardiotis E. Prevalence and determinants of subjective cognitive decline in a representative Greek elderly population. Int J Geriatr Psychiatry. (2019) 34:846–54. doi: 10.1002/gps.5073, PMID: 30714214

[27] Lee SH Kang Y Cho SJ. Subjective cognitive decline in patients with migraine and its relationship with depression, anxiety, and sleep quality. J Headache Pain. (2017) 18:77. doi: 10.1186/s10194-017-0779-1, PMID: 28744704 PMC5526827

[28] Valtorta N Hanratty B. Loneliness, isolation, and the health of older adults: do we need a new research agenda? J R Soc Med Suppl. (2012) 105:518–22. doi: 10.1258/jrsm.2012.120128, PMID: 23288086 PMC3536512

[29] Lara E Martín-María N De la Torre-Luque A. Does loneliness contribute to mild cognitive impairment and dementia? A systematic review and meta-analysis of longitudinal studies. Ageing Res Rev. (2019) 52:7–16. doi: 10.1016/j.arr.2019.03.002, PMID: 30914351

[30] Perissinotto CM Stijacic Cenzer I Covinsky KE. Loneliness in older persons: a predictor of functional decline and death. Arch Intern Med. (2012) 172:1078–83. doi: 10.1001/archinternmed.2012.1993, PMID: 22710744 PMC4383762

[31] Valtorta NK Kanaan M Gilbody S Hanratty B. Loneliness, social isolation, and risk of cardiovascular disease in the English longitudinal study of ageing. Eur J Prev Cardiol. (2018) 25:1387–96. doi: 10.1177/2047487318792696, PMID: 30068233

[32] Elovainio M Hakulinen C Pulkki-Råback L Virtanen M Josefsson K Jokela M. Contribution of risk factors to excess mortality in isolated and lonely individuals: an analysis of data from the UK biobank cohort study. Lancet Public Health. (2017) 2:e260–6. doi: 10.1016/S2468-2667(17)30075-0, PMID: 28626828 PMC5463031

[33] Zhang X Ma Q Cao R Huang X Ming Z Liu Y. Meta-analysis of risk factors for subjective cognitive decline in community elderly. Chin J Nurs. (2023) 58:342–8.

[34] Livingston G Huntley J Liu KY Costafreda SG Selbæk G Alladi S. Dementia prevention, intervention, and care: 2024 report of the lancet standing commission. Lancet. (2024) 404:572–628. doi: 10.1016/S0140-6736(24)01296-0, PMID: 39096926

[35] Gifford KA Liu D Romano R Jones RN Jefferson AL Jianguo J. Development of a subjective cognitive decline questionnaire using item response theory: a pilot study. Alzheimers Dement (Amst). (2015) 1:429–39. doi: 10.1016/j.dadm.2015.09.004, PMID: 26878034 PMC4750048

[36] Lixiao H Xiaochen H Ying H. Localization of subjective cognitive decline questionnaire and its reliability and validity test. Chin Gen Pract. (2019) 22:3238–45.

[37] Fried LP Tangen CM Walston J Newman AB Hirsch C Gottdiener J. Frailty in older adults: evidence for a phenotype. J Gerontol A Biol Sci Med Sci. (2001) 56:M146–56. doi: 10.1093/gerona/56.3.m146, PMID: 11253156

[38] Ran G Wang Y Liu S Liu D. Multidimensional social deprivation impacts on frailty in the elderly: The mediating effect of depression. Sichuan Da Xue Xue Bao Yi Xue Ban. (2024) 55:925–31. doi: 10.12182/20240760601, PMID: 39170020 PMC11334278

[39] Hoyl MT Alessi CA Harker JO Josephson KR Pietruszka FM Koelfgen M. Development and testing of a five-item version of the geriatric depression scale. J Am Geriatr Soc. (1999) 47:873–8. doi: 10.1111/j.1532-5415.1999.tb03848.x, PMID: 10404935

[40] Liu CC Chen LQ Xie BQ Chen Y Wang JL Xu HQ. Prevalence and determinants of social isolation among community-living oldest-old adults. Nurs J. (2022) 37:98–102.

[41] Lubben J Blozik E Gillmann G Iliffe S von Renteln Kruse W Beck JC. Performance of an abbreviated version of the Lubben social network scale among three European community-dwelling older adult populations. Gerontologist. (2006) 46:503–13. doi: 10.1093/geront/46.4.503, PMID: 16921004

[42] Sturm N Krisam J Szecsenyi J Bentner M Frick E Mächler R. Spirituality, self-care, and social activity in the primary medical care of elderly patients. Dtsch Arztebl Int. (2022) 119:124–31. doi: 10.3238/arztebl.m2022.0078, PMID: 34939916 PMC9160422

[43] Rubenstein LZ Harker JO Salvà A Guigoz Y Vellas B. Screening for undernutrition in geriatric practice: developing the short-form mini-nutritional assessment (MNA-SF). J Gerontol A Biol Sci Med Sci. (2001) 56:M366–72. doi: 10.1093/gerona/56.6.M366, PMID: 11382797

[44] Ning H Du Y Ellis D Deng HW Hu H Zhao Y. Malnutrition and its associated factors among elderly Chinese with physical functional dependency. Public Health Nutr. (2021) 24:1404–14. doi: 10.1017/S1368980019005299, PMID: 32389160 PMC7864553

[45] Cheng GR Liu D Huang LY Han GB Hu FF Wu ZX. Prevalence and risk factors for subjective cognitive decline and the correlation with objective cognition among community-dwelling older adults in China: results from the Hubei memory and aging cohort study. Alzheimers Dement. (2023) 19:5074–85. doi: 10.1002/alz.13047, PMID: 37186161

[46] Liew TM. Depression, subjective cognitive decline, and the risk of neurocognitive disorders. Alzheimer's Res Ther. (2019) 11:70. doi: 10.1186/s13195-019-0527-7, PMID: 31399132 PMC6689179

[47] Lauriola M Esposito R Delli Pizzi S de Zambotti M Londrillo F Kramer JH. Sleep changes without medial temporal lobe or brain cortical changes in community-dwelling individuals with subjective cognitive decline. Alzheimers Dement. (2017) 13:783–91. doi: 10.1016/j.jalz.2016.11.006, PMID: 28034600 PMC5749240

[48] Xu S Ren Y Liu R Li Y Hou T Wang Y. Prevalence and progression of subjective cognitive decline among rural Chinese older adults: a population-based study. J Alzheimer's Dis. (2023) 93:1355–68. doi: 10.3233/JAD-221280, PMID: 37182880

[49] Xue C Li J Fang Q Hao MQ Chen LH Chen ZX. Meta-analysis of the prevalence of subjective cognitive decline among elderly Chinese. J Practical Cardio-Cerebrovascular Dis. (2023) 31:67–72.

[50] Wang NF Song ZY Liu XL Wang LY Cao JX Du YF. Characteristics and influencing factors of subjective cognitive decline among rural elderly. Chin J Behav Med Sci. (2021) 30:402–7.

[51] Liu S Yuan X Liang H Jiang Z Yang X Gao H. Development and validation of frailty risk prediction model for elderly patients with coronary heart disease. BMC Geriatr. (2024) 24:742. doi: 10.1186/s12877-024-05320-7, PMID: 39244543 PMC11380413

[52] Robertson DA Savva GM Kenny RA. Frailty and cognitive impairment: a review of the evidence and causal mechanisms. Ageing Res Rev. (2013) 12:840–51. doi: 10.1016/j.arr.2013.06.004, PMID: 23831959

[53] Huang J Zeng X Ning H Peng R Guo Y Hu M. Development and validation of prediction model for older adults with cognitive frailty. Aging Clin Exp Res. (2024) 36:8. doi: 10.1007/s40520-023-02647-w, PMID: 38281238 PMC10822804

[54] Stephan Y Sutin AR Luchetti M Terracciano A. Feeling older and the development of cognitive impairment and dementia. J Gerontol B Psychol Sci Soc Sci. (2017) 72:966–73. doi: 10.1093/geronb/gbw085, PMID: 27436103 PMC5927095

[55] Wang YX Fu R Zhang JW Niu QX Li CH Liang LP. Meta-analysis of the risk factors for cognitive frailty in community-dwelling elderly. Chongqing Med. (2022) 51:3364–9.

[56] Lin F Liu SZ Jiang HY. Analysis of factors influencing cognitive frailty in elderly patients with ischemic stroke. Zhejiang Clin Med. (2023) 25:1012–4.

[57] Seesen M Sirikul W Ruangsuriya J Griffiths J Siviroj P. Cognitive frailty in Thai community-dwelling elderly: prevalence and its association with malnutrition. Nutrients. (2021) 13:4239. doi: 10.3390/nu13124239, PMID: 34959791 PMC8709040

[58] Zhang Z Wei F Shen XN Ma YH Chen KL Dong Q. Associations of subsyndromal symptomatic depression with cognitive decline and brain atrophy in elderly individuals without dementia: a longitudinal study. J Affect Disord. (2020) 274:262–8. doi: 10.1016/j.jad.2020.05.097, PMID: 32469814

[59] Zhou L Ma X Wang W. Relationship between cognitive performance and depressive symptoms in Chinese older adults: The China health and retirement longitudinal study (CHARLS). J Affect Disord. (2021) 281:454–8. doi: 10.1016/j.jad.2020.12.059, PMID: 33360747

[60] Huijun L Xiange Z Ming Y Jiayi S Juanjuan P Wangquan X. The mediating role of daily living ability and sleep in depression and cognitive function based on a structural equation model. BMC Geriatr. (2025) 25:223. doi: 10.1186/s12877-025-05871-3, PMID: 40186110 PMC11971737

[61] Price RB Duman R. Neuroplasticity in cognitive and psychological mechanisms of depression: an integrative model. Mol Psychiatry. (2020) 25:530–43. doi: 10.1038/s41380-019-0615-x, PMID: 31801966 PMC7047599

[62] Rapp MA Schnaider-Beeri M Grossman HT Sano M Perl DP Purohit DP. Increased hippocampal plaques and tangles in patients with Alzheimer disease with a lifetime history of major depression. Arch Gen Psychiatry. (2006) 63:161–7. doi: 10.1001/archpsyc.63.2.161, PMID: 16461859

[63] Ménard C Hodes GE Russo SJ. Pathogenesis of depression: insights from human and rodent studies. Neuroscience. (2016) 321:138–62. doi: 10.1016/j.neuroscience.2015.05.053, PMID: 26037806 PMC4664582

[64] Wang JH. Cohort study on the changes in dietary diversity scores and their relationship with cognitive impairment and activities of daily living dependency in elderly Chinese[D]. Guangzhou: Southern Medical University (2022).

[65] Hsu YH Chou MY Chu CS Liao MC Wang YC Lin YT. Predictive effect of malnutrition on long-term clinical outcomes among older men: a prospectively observational cohort study. J Nutr Health Aging. (2019) 23:876–82. doi: 10.1007/s12603-019-1246-2, PMID: 31641739 PMC12280699

[66] Yeh TS Yuan C Ascherio A Rosner BA Blacker D Willett WC. Long-term dietary protein intake and subjective cognitive decline in US men and women. Am J Clin Nutr. (2022) 115:199–210. doi: 10.1093/ajcn/nqab236, PMID: 34293099 PMC8755047

[67] Yeh TS Yuan C Ascherio A Rosner BA Willett WC Blacker D. Long-term dietary flavonoid intake and subjective cognitive decline in US men and women. Neurology. (2021) 97:e1041–56. doi: 10.1212/WNL.0000000000012454, PMID: 34321362 PMC8448553

[68] Guarnieri L Bosco F Leo A Citraro R Palma E de Sarro G. Impact of micronutrients and nutraceuticals on cognitive function and performance in Alzheimer’s disease. Ageing Res Rev. (2024) 95:102210–34. doi: 10.1016/j.arr.2024.102210, PMID: 38296163

[69] O’Connor D Molloy AM Laird E Kenny RA O’Halloran AM. Sustaining an ageing population: the role of micronutrients in frailty and cognitive impairment. Proc Nutr Soc. (2023) 82:315–28. 36938798 10.1017/S0029665123002707

[70] Lee SL Thomas P Fenech M. Genome instability biomarkers and blood micronutrient risk profiles associated with mild cognitive impairment and Alzheimer’s disease. Mutat Res. (2015) 776:54–83. doi: 10.1016/j.mrfmmm.2014.12.012, PMID: 26364206

[71] Simo-Tabue N Boucaud-Maitre D Letchimy L Guilhem-Decleon J Helene-Pelage J Duval GT. Correlates of undernutrition in older people in Guadeloupe (French West Indies): results from the KASADS study. Nutrients. (2023) 15:2950. doi: 10.3390/nu15132950, PMID: 37447276 PMC10346391

[72] Mengel D Soter E Ott JM Wacker M Leyva A Peters O. Blood biomarkers confirm subjective cognitive decline (SCD) as a distinct molecular and clinical stage within the NIA-AA framework of Alzheimer’s disease. Mol Psychiatry. (2025) 30:3150–9. doi: 10.1038/s41380-025-03021-0, PMID: 40247130 PMC12185333

[73] Albala C Lera L Sanchez H Angel B Márquez C Arroyo P. Frequency of frailty and its association with cognitive status and survival in older Chileans. Clin Interv Aging. (2017) 12:995–1001. doi: 10.2147/CIA.S136906, PMID: 28721027 PMC5498773

[74] Wang Y Zhou MF Chen SX Zhu YP Pan YJ Wang Y. Study on subjective cognitive decline and its correlation with frailty in peritoneal dialysis patients. J Mil Nurs. (2023) 40:49–52.

[75] Ma L Zhang L Sun F Li Y Tang Z. Cognitive function in prefrail and frail community-dwelling older adults in China. BMC Geriatr. (2019) 19:53. doi: 10.1186/s12877-019-1056-8, PMID: 30813907 PMC6391822

[76] Yuan Y Lapane KL Tjia J Baek J Liu SH Ulbricht CM. Trajectories of physical frailty and cognitive impairment in older adults in United States nursing homes. BMC Geriatr. (2022) 22:339. doi: 10.1186/s12877-022-03012-8, PMID: 35439970 PMC9017032

[77] Peng C Burr JA Yuan Y Lapane KL. Physical frailty and cognitive function among older Chinese adults: The mediating roles of activities of daily living limitations and depression. J Frailty Aging. (2023) 12:156–65. doi: 10.14283/jfa.2023.1, PMID: 37493375 PMC10372340

[78] Yu R Morley JE Kwok T Leung J Cheung O Woo J. The effects of combinations of cognitive impairment and pre-frailty on adverse outcomes from a prospective community-based cohort study of older Chinese people. Front Med (Lausanne). (2018) 5:50. doi: 10.3389/fmed.2018.00050, PMID: 29600247 PMC5863513

[79] Li C Ge S Yin Y Tian C Mei Y Han P. Frailty is associated with worse cognitive functioning in older adults. Front Psych. (2023) 14:1108902. doi: 10.3389/fpsyt.2023.1108902, PMID: 36816402 PMC9928943

[80] Koszalinski RS Olmos B. Communication challenges in social isolation, subjective cognitive decline, and mental health status in older adults: a scoping review (2019-2021). Perspect Psychiatr Care. (2022) 58:2741–55. doi: 10.1111/ppc.13115, PMID: 35582750

[81] Yang JM Lee HJ Kim JH. Association between social isolation and subjective cognitive decline in Korean older adult population: a nationwide cross-sectional study in South Korea. Prev Med Rep. (2023) 34:102261. doi: 10.1016/j.pmedr.2023.102261, PMID: 37387723 PMC10302111

[82] Wu GL Zhang L Zhao YX. The impact of social isolation and sleep quality, and their interaction on cognitive function in elderly people. J Pract Geriatr. (2023) 37:495–9.

[83] Wrzus C Hänel M Wagner J Neyer FJ. Social network changes and life events across the life span: a meta-analysis. Psychol Bull. (2013) 139:53–80. doi: 10.1037/a0028601, PMID: 22642230

[84] Kim YB. The study on relationship between social isolation and cognitive function in elderly Korean. J Digit Converg. (2018) 16:429–39.

[85] Jang Y Choi EY Park NS Chiriboga DA Duan L Kim MT. Cognitive health risks posed by social isolation and loneliness in older Korean Americans. BMC Geriatr. (2021) 21:123. doi: 10.1186/s12877-021-02066-4, PMID: 33593273 PMC7885241

[86] Livingston G Huntley J Sommerlad A Ames D Ballard C Banerjee S. Dementia prevention, intervention, and care: 2020 report of the lancet commission. Lancet. (2020) 396:413–46. doi: 10.1016/S0140-6736(20)30367-6, PMID: 32738937 PMC7392084

[87] Hao XY Li CS Wang XH Zhan TX. Study on the recognition of cognitive function trajectories and the impact of lifestyle in elderly individuals. Nurs J. (2023) 38:103–8.

[88] Tang R Yuan MQ Han YF Fang Y. Quantile regression analysis of the impact of depression and social interaction on cognitive function in elderly people. Chin J Health Stat. (2022) 39:397–9.

[89] He HH. Study on the impact of bidirectional intergenerational support on cognitive function in elderly people[D]. Yangzhou: Yangzhou University (2023).

